# Inhibition of epileptiform activity by neuropeptide Y in brain tissue from drug-resistant temporal lobe epilepsy patients

**DOI:** 10.1038/s41598-019-56062-1

**Published:** 2019-12-18

**Authors:** Jenny Wickham, Marco Ledri, Johan Bengzon, Bo Jespersen, Lars H. Pinborg, Elisabet Englund, David P. D. Woldbye, My Andersson, Merab Kokaia

**Affiliations:** 1 Epilepsy Centre, Department of Clinical Sciences, Lund University, Lund University Hospital, SE-22362 Lund, Sweden; 2 Neurosurgery, Department of Clinical Sciences, Lund University, Skånes Universitetssjukhus, SE-22362 Lund, Sweden; 3 Department of Neurosurgery, Rigshospitalet, Copenhagen University Hospital, University of Copenhagen, DK-2100 Copenhagen, Denmark; 4 Department of Neurology and Neurobiology Research Unit, Copenhagen University Hospital, Rigshospitalet, Building 6931, Blegdamsvej 9, DK-2100 Copenhagen, Denmark; 5 Division of Oncology and Pathology, Department of Clinical Sciences, Lund University, SE-22362 Lund, Sweden; 6 Laboratory of Neuropsychiatry, Department of Neuroscience, University of Copenhagen, DK-2100 Copenhagen, Denmark

**Keywords:** Epilepsy, Translational research

## Abstract

In epilepsy patients, drug-resistant seizures often originate in one of the temporal lobes. In selected cases, when certain requirements are met, this area is surgically resected for therapeutic reasons. We kept the resected tissue slices alive *in vitro* for 48 h to create a platform for testing a novel treatment strategy based on neuropeptide Y (NPY) against drug-resistant epilepsy. We demonstrate that NPY exerts a significant inhibitory effect on epileptiform activity, recorded with whole-cell patch-clamp, in human hippocampal dentate gyrus. Application of NPY reduced overall number of paroxysmal depolarising shifts and action potentials. This effect was mediated by Y2 receptors, since application of selective Y2-receptor antagonist blocked the effect of NPY. This proof-of-concept finding is an important translational milestone for validating NPY-based gene therapy for targeting focal drug-resistant epilepsies, and increasing the prospects for positive outcome in potential clinical trials.

## Introduction

Epilepsy is a multifactorial neurological disease characterised by increased excitability in the brain, leading to pathological hyper-synchronised activity of neurons manifested as spontaneous recurrent seizures (SRS). In a majority of cases, the symptomatic treatment by currently available anti-epilepsy drugs (AEDs) eliminates SRS under the medication period^1^. However, AEDs have unwanted side effects, and most importantly, are ineffective in about 30–40% of epilepsy patients^2,3^. The temporal lobe is one of the most common structures for focal seizure origin, and about a third of these patients are AED resistant^1^. These patients are considered for resection of the epileptic tissue, provided the seizure-generating focal area is reliably identified and its removal poses no substantial risk for post-operative functional deficit. Once resected, the temporal lobe is taken for histopathological examination. The therapeutic surgery also provides a unique opportunity to use parts of the resected epileptic tissue for functional studies *in vitro*, utilizing various electrophysiological methodologies. These studies help in understanding pathophysiological mechanisms of ictogenesis and allow for testing of novel treatment strategies in human epileptic tissue^4–6^.

**Figure 1:**
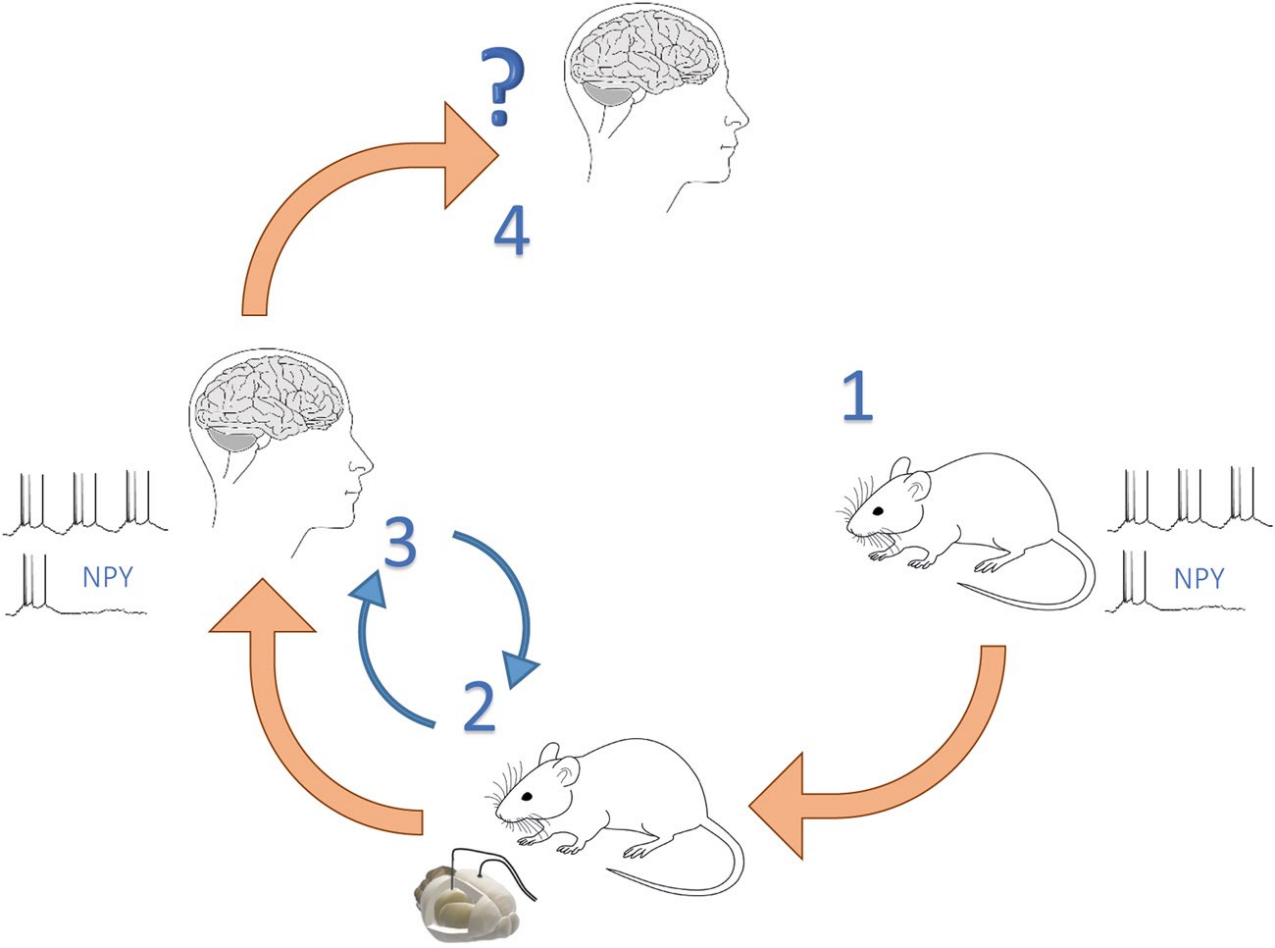
Translational roadmap for the use of human brain tissue as a step between animal research and clinical trials. Schematic illustration of how human-tissue resected from patients with epilepsy can be used to validate the effect of treatments found in animal models before proceeding to clinical trials for drug-resistant epilepsy, exemplified by NPY. (1) Basic research, with *in vitro* models, from several labs has shown that NPY has an inhibitory effect on epileptiform activity. (2) Chronic epilepsy models *in vivo*, such as the intrahippocampal kainate model, has provided key evidence demonstrating that NPY has an anti-epileptic effect in the chronic epileptic brain over a longer period of time. (3) The present study is a crucial validation step showing that NPY has an inhibitory effect on epileptiform activity in the target tissue, drug-resistant human hippocampal slices, minimising the risk for the next step of first-in-man clinical phase 1–2 study (step 4). The step 2 is interchangeable with step 3.

Neuropeptide Y (NPY) is a 36 amino acid long peptide^7^ widely expressed in the brain and released from presynaptic terminals mostly during high-frequency activity^8,9^. Overexpression of NPY has been shown to be highly effective at suppressing epileptic activity in various animal models of both acute and chronic seizures^10–14^ (Fig. 1). This effect of NPY on seizures is thought to occur through the activation of presynaptic NPY Y2 receptors^11,15,16^ that inhibit voltage gated calcium channels^17^, thereby reducing glutamate release probability in excitatory synapses^18,19^. The reduction in glutamate release during NPY application have also been documented in dentate granule cells during electrical stimulation of the lateral perforant path in human brain slices^20^. Previously, we have also shown that Y2 receptors are present in human hippocampal tissue resected from temporal lobe of drug-resistant epilepsy patients and that exogenous application of NPY decrease basal synaptic transmission in these slices through activation of Y2 receptors^6^. This and other studies suggested that gene therapy based on overexpression of NPY alone or in combination with Y2 receptors might provide an alternative treatment strategy for drug-resistant temporal lobe epilepsy (TLE)^6,11,21^. Although it has been shown that NPY application reduces glutamate release in human drug-resistant epileptic tissue^6,20^, an important step toward a clinical gene therapy trial (Fig. 1, step 3), namely direct evidence that NPY can also suppress seizure-like activity in human hippocampal slices, is still lacking.

We addressed this question by inducing epileptiform seizure-like activity in human hippocampal slices while performing whole-cell patch-clamp recordings in granule cells of the dentate gyrus and tested the inhibitory effect of NPY on this activity.

We demonstrate, for the first time, that NPY effectively suppresses epileptiform discharges in human drug-resistant hippocampal slices, providing direct evidence that increased NPY signalling in human epileptic tissue is capable of inhibiting seizure-like activity.

## Results

### Resected human hippocampal tissue

**Figure 2:**
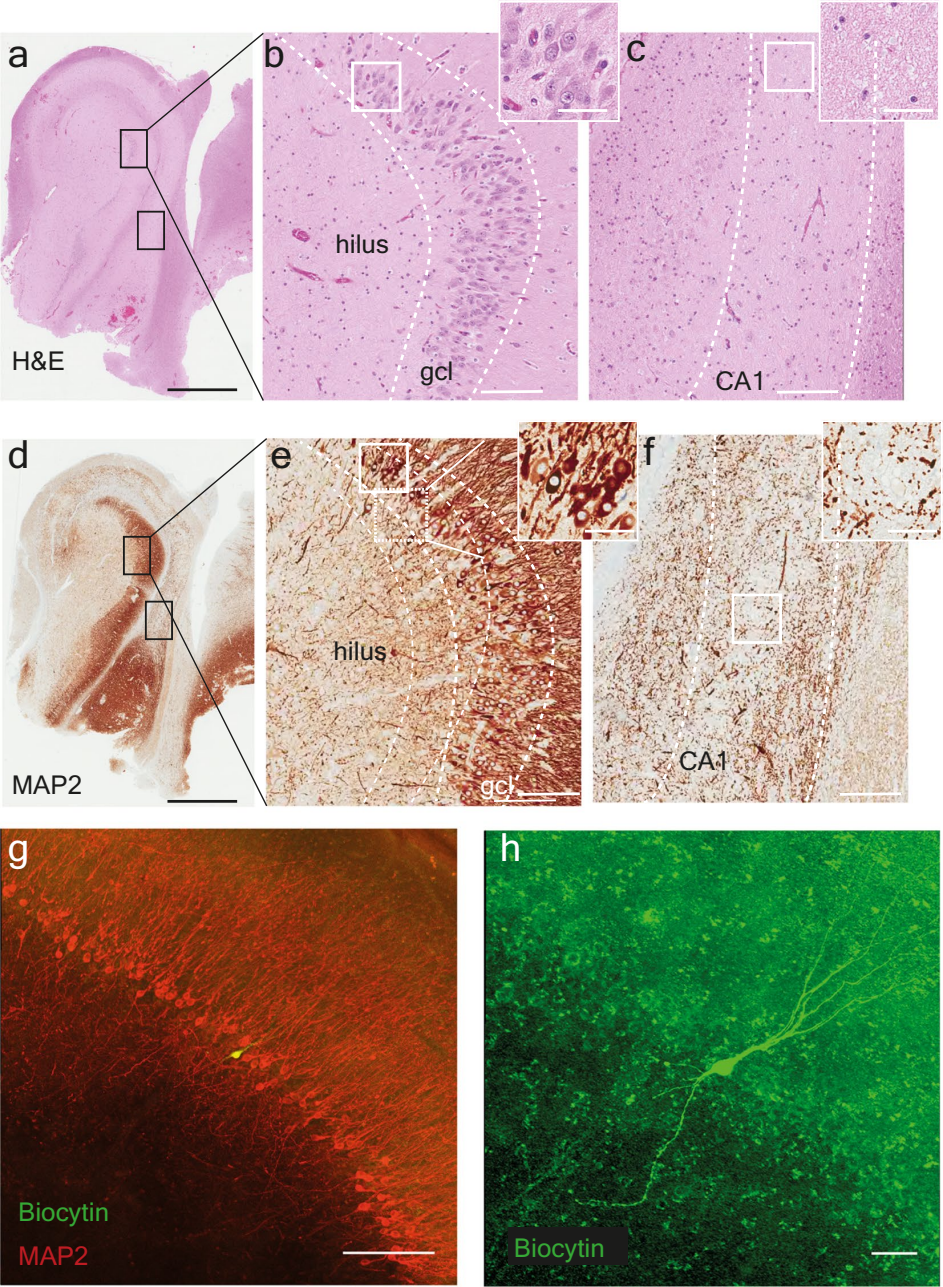
Neuropathological evaluation and granule cell layer with biocytin-filled cell: Example of (**a–c**) H&E and (**d–f**) MAP2 staining in hippocampal tissue resected from a patient with TLE. The sectioning and staining are made for neuropathological evaluation showing (**a,d**) the whole slice including dentate gyrus, CA3-CA1 and subiculum. The electrophysiological experiments were performed in the dentate gyrus magnified in B and E with the granule cell layer (gcl) identified, and further magnification inset showing individual granule cells. (**c,f**) In CA1, the neuronal cell loss is clear, the pyramidal cell layer has completely disappeared. After electrophysiological experiments the slices were fixated in PFA and later stained for (**g**) MAP2 (red) showing the granule cell layer with the recorded biocytin-filled granule cell (in green). (**h**) Higher magnification of the recorded neuron with typical granule cell morphology. Scale bar: 2 mm in a and d, 150 µm in b, c, e and f with 50 µm in inset, 200 µm in g and 50 µm in h.

Hippocampal tissue samples from ten patients undergoing surgery for drug-resistant TLE were collected for this study with their medical characteristics summarized in Table 1. Neuropathological examination of resected tissue with respect to hippocampal sclerosis and other pathologies were performed on hematoxylin-eosin- (H&E, Fig. 2a–c) and microtubule-associated protein 2- (MAP2, Fig. 2d–f) stained paraffin sections. The majority of resected tissue displayed hippocampal sclerosis (Table 1). Various degrees of degeneration were observed in the dentate gyrus (Fig. 2b,e) and in CA1 areas (Fig. 2c,f). Neuronal loss was particularly pronounced in the CA1 pyramidal cell layer. Within the dentate gyrus, neuronal cell loss was seen as a thinning of the granule cell layer towards both the hilus and molecular layer. No oedema was detected, indicating that these changes did not occur acutely, in the immediate time period prior to resection.

**Table 1:** Patient Data.

Patient	Resistance to >2 AEDs	Age at surgery (yrs)	Duration of epilepsy (from onset; yrs)	Seizure frequency (n/mo)	AEDs at surgery	Hippocampal pathology
1	yes	31	12	1 (SFS) 1 (CFS)	LTG, LEV	HS
2	yes	57	5	9 (CFS)	OXC	HS
3	yes	34	8	3 (CFS)	CBZ, LEV, LAC	HS
4	yes	19	13	6 (SFS) 6 (CFS)	LEV, CBZ	HS
5	yes	23	22	4 (CFS)	LEV, LAC	HS
6	yes	27	8	25 approx.	LAM, FRI, VAL	Neuronal loss and gliosis
7	yes	44	7	5 (CFS)	LTG, ZNS, BRI	Normal
8	yes	24	10	20 (CFS)	LAC	HS
9	yes	42	6	2 (CFS)	CLB, LAC, ZNS	HS
10	yes	38	35	5 (CFS)	FEN, LTG, LEV, LAC	HS

Seizure frequency reported by patients in Copenhagen (CPH) as simple focal seizures (SFSs) and complex focal seizures (CFSs). Abbreviations are as follows: carbamazepine Brivaracetam (BV), (CBZ), clobazam (CLB), Fenytoin (FEN) hippocampal sclerosis (HS), lacosamide (LAC), levetiracetam (LEV), lamotrigine (LTG), oxcarbazepine (OXC), valproate (VPA) and zonesamide (ZNS).

### NPY increases inter-spike interval, decreases number of paroxysmal depolarising shifts and the total number of action potentials

After 24 hours of incubation in the interface chamber, the slices were individually transferred to the dual-flow recording chamber. After acquiring whole-cell patch-clamp configuration of the dentate granule cell, resting membrane potential (RMP) was measured in continuous current-clamp mode to an average of −67.22 ± 1.16 mV (Table 2). RMP, intrinsic properties of the recorded cells (Table 2), their morphology and position in the dentate gyrus (Fig. 2a,b) confirmed that they were granule cells.

**Table 2:** Intrinsic properties.

	All cells (n = 20)	NPY (n = 11)	NPY + BIIE0246 (n = 9)
RPM (mV)	−67.22 ± 1.16	−68.72 ± 1.06	−65.39 ± 2.16
Input R (Ω)	166.3 ± 38.18	161.2 ± 16.57	172.4 ± 78.89
AP threshold (mV)	−38.18 ± 1.10	−39.43 ± 1.20	−36.65 ± 1.92
AP half-width (ms)	0.74 ± 0.03	0.73 ± 0.05	0.74 ± 0.01
AP amplitude (mV)	84.69 ± 1.77	88.91 ± 2.18	79.53 ± 1.81

**Figure 3:**
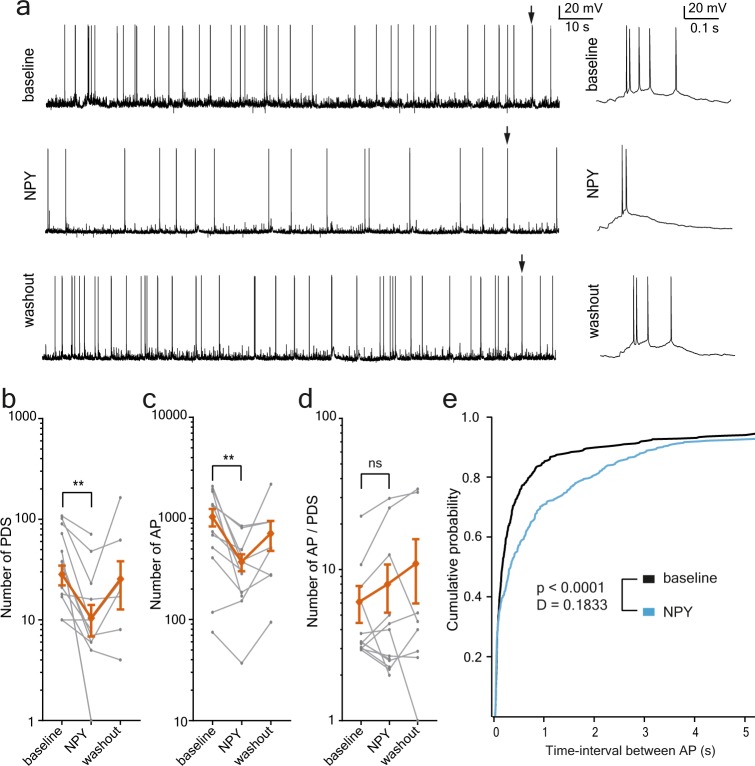
NPY-application suppresses epileptiform activity in human drug-resistant hippocampal slices. (**a**) Example recordings from dentate granule cells during epileptiform activity triggered by 0Mg^2+^/4-AP aCSF. Whole-cell recordings in current-clamp mode, arrows indicate the PDSs that are displayed in expanded time-scale to the right of each sweep. NPY application resulted in a marked reduction of APs and PDS frequencies compared to the *baseline*, and partial return to the *baseline* levels during the washout period. (**b–d**) The average values (±SEM) are displayed in orange, the individual values – in grey. (**b**) The number of PDS was reduced during the application of NPY (*baseline*: 47.42 ± 10.52 and *NPY*: 17.6 ± 6.06, n = 12, t-test, p = 0.0022). (**c**) The number of APs was reduced during the application of NPY (*baseline*: 1042 ± 205.1 and *NPY*: 371.8 ± 71.49, n = 12, t-test, p = 0.0013). (**d**) The number of APs during individual PDSs did not change during the application of NPY (*baseline*: 6.067 ± 1.66 and *NPY*: 7.94 ± 2.786, n = 12, t-test, p = 0.9436). (**e**) Cumulative plot of the time-interval between APs in baseline recordings (black) and recordings during NPY application (blue). A significant difference between *baseline* and *NPY* (p < 0.0001, D = 0.1833) was detected with the Kolmogorov-Smirnov test.

**Figure 4:**
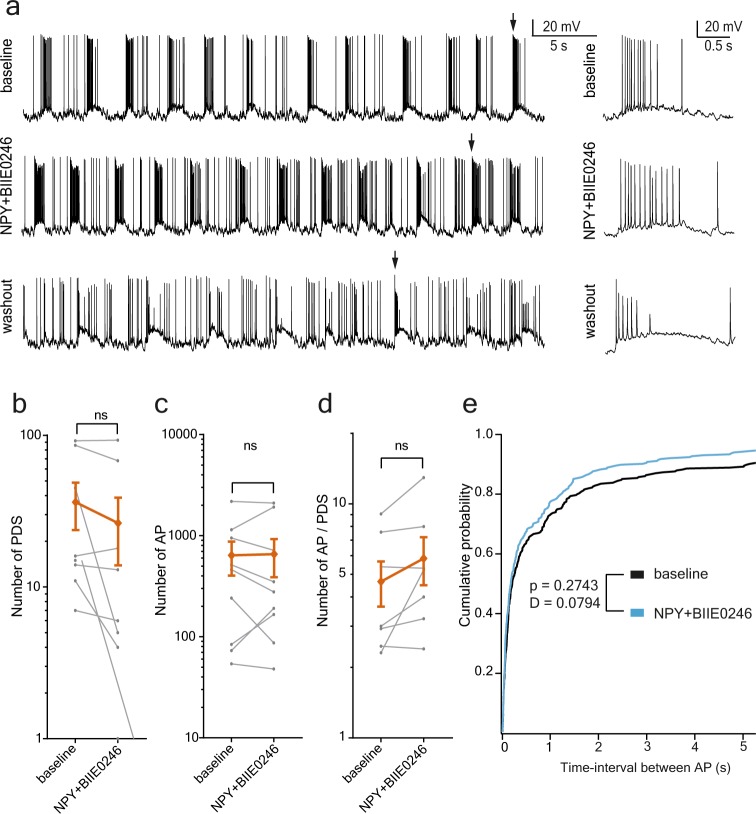
NPY application together with its Y2-receptor antagonist BIIE0246 has no effect on epileptiform activity. (**a**) Example recordings from dentate granule cells during epileptiform activity triggered by 0Mg^2+^/4-AP aCSF. Whole-cell recordings in current-clamp mode, arrows indicate the PDSs that are displayed in zoomed-in time-scale to the right of each sweep. NPY applied together with the Y2 receptor antagonist BIIE0246; under these conditions a reduction in AP or PDS frequency is not observed. (**b–d**) The average values (±SEM) are displayed in orange, the individual values – in grey. (**b**) The number of PDS is unchanged during the application of NPY + BIIE0246 (*baseline*: 35.75 ± 12.33 and *NPY* + *BIIE0246*: 26 ± 12.27, n = 8, t-test, p = 0.0834). (**c**) The number of APs is unchanged during the application of NPY + BIIE0246 (*baseline*: 633.6 ± 232.8 and *NPY* + *BIIE0246*: 650.4 ± 265.3, n = 9, t-test, p = 0.874). (**d**) The number of APs during the individual PDSs is unchanged during the application of NPY + BIIE0246 (*baseline*: 4.674 ± 1.028 and *NPY* + *BIIE0246*: 5.873 ± 1.361, n = 7, t-test, p = 0.094). (**e**) Cumulative plot of the time-interval between APs during the baseline recordings (black) and recordings during NPY + BIIE0246 application (blue). No difference between *baseline* and *NPY* + *BIIE0246* (p = 0.2743, D = 0.0794) was detected with the Kolmogorov-Smirnov test.

[0Mg^2+^]/4-aminopyridine (4-AP) – artificial cerebrospinal fluid (aCSF) solution was bath applied until stable epileptiform activity over several minutes was achieved, as judged by the appearance of regular paroxysmal depolarising shifts (PDS) in granule cells (Fig. 3a). Subsequently, NPY-[0Mg^2+^]/4-AP-aCSF was applied during 10 min, followed by a 10–30 min period of wash-out without NPY (Fig. 3a). To assess the possible effect of NPY on epileptiform activity, we measured (i) the time between action potentials (AP, inter-spike intervals), (ii) the number of PDS-events, (iii) the total number of APs and (iv) the average number of APs during individual PDS-events. These parameters were compared before, during and after NPY (Fig. 3) or NPY + BIIE0246 (Fig. 4) application (termed as *baseline, NPY, NPY* + *BIIE0246* and *washout* periods, respectively). The results demonstrate that there was a significant increase in inter-spike intervals during NPY application, as measured by the Kolmogorov-Smirnov test of the cumulative probability (summarised cumulative probability plot of inter-spike interval: comparing *baseline, n* = *12*, with *NPY, n* = *12*, p < 0.0001, D = 0.1833, Kolmogorov-Smirnov test, Fig. 3e).

We also observed a 60% reduction in the number of PDS-events during NPY application as compared to *baseline* (during *baseline* 47.4 ± 10.52 and *NPY* 17.5 ± 6.06, t(11) = 3.974, p = 0.0022. *n* = *12*, t-test, Fig. 3b). Similarly, the total number of APs during the whole 300 s long analysis period was significantly decreased by 60% during *NPY* application as compared to *baseline* (during *baseline* 1042 ± 205.1 and *NPY* 371.8 ± 71.49, t(11) = 4.295, p = 0.0013. *n* = *12*, t-test, Fig. 3c). In a subset of the cells, with successful recordings long enough to monitor the washout period, the number of PDS-events returned to *baseline* levels during washout (during *baseline* 44.3 ± 13.94 and *washout* 42.7 ± 21.44, t(6) = 1.115, p = 0.3075. *n* = *7*, t-test, Fig. 3b). Similarly, the total number of APs was not different between *baseline* and *washout* (during *baseline* 853.5 ± 246.8 and *washout* 712.8 ± 235.1, t(7) = 0.2314, p = 0.8236. *n* = *8*, t-test, Fig. 3c). In contrast, the average number of APs during each PDS-events was not significantly altered by *NPY* treatment (during *baseline* 6.1 ± 1.66 and *NPY* 7.9 ± 2.79, t(11) = 0.07241, p = 0.9436. *n* = *12*, t-test, Fig. 3d).

### Application of Y2 receptor antagonist blocks the effect of NPY

We hypothesised that the observed effect of NPY was due to decreased glutamate release from presynaptic terminals by activation of presynaptic Y2 receptors^6,20^. To address this question, NPY was applied together with the Y2 receptor antagonist BIIE0246 in a set of experiments (Fig. 4a). No significant difference could be detected in the inter-spike intervals during *baseline* and NPY + BIIE0246 application (summarised cumulative probability plot of inter-spike interval: comparing *baseline, n* = *9*, with *NPY* + *BIIE0246*, *n* = *9*, p < 0.2743, D = 0.0794, Kolmogorov-Smirnov test, Fig. 4e). Similarly, the number of PDS-events (during *baseline* 35.8 ± 12.33 and *NPY* + *BIIE0246* 26 ± 12.27, t(7) = 2.018, p = 0.0834. *n* = *8*, t-test, Fig. 4b) or the number of AP (during *baseline* 633.6 ± 232.8 and *NPY* + *BIIE0246* 650.4 ± 265.3, t(8) = 0.1637, p = 0.874. *n* = *9*, t-test, Fig. 4c) did not differ significantly between *baseline* and NPY + BIIE0246 application. The number of AP in each PDS was also not different from *baseline* (during *baseline* 4.7 ± 1.03 and *NPY* + *BIIE0246* 5.87 ± 1.36, t(6) = 1.988, p = 0.094. n = 7, t-test, Fig. 4d).

**Figure 5:**
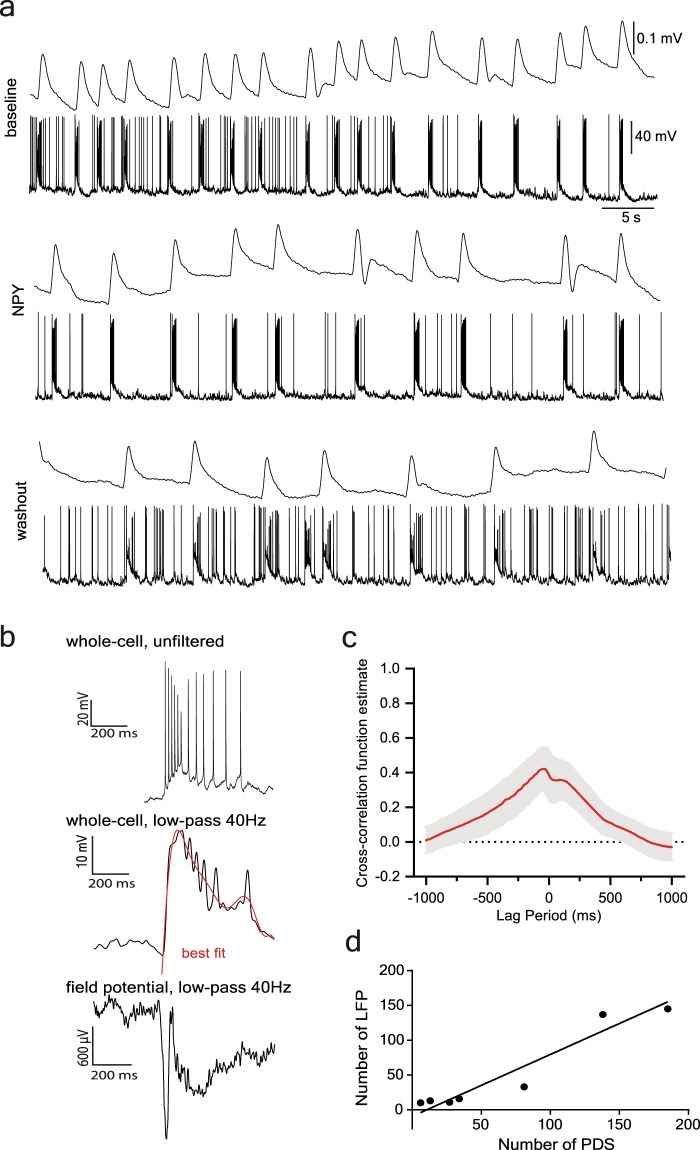
Simultaneous whole-cell current-clamp and field-recordings in the dentate gyrus during NPY application. (**a**) Representative recordings showing simultaneous whole-cell current-clamp recording and a field recording during baseline recording, NPY application, and washout period. Whole-cell recording from individual granule cell demonstrating 4-AP induced PDSs that are time-locked with field potential bursting. Both PDS and corresponding field potential bursting showed parallel reductions in frequency during NPY application. (**b**) Representative recordings showing an unfiltered PDS (top), a PDS low-pass filtered at 40 Hz with the best fit indicated by the red line (middle) and a low-pass filtered at 40 Hz field potential (bottom). (**c**) Cross-correlation analysis of whole-cell and field recordings showing a significant degree of time correlation, with the cross-correlation function estimate peaking close to 0 ms. The red trace is the average correlation estimate across recordings, the grey shadow represents SEM. (**d**) The number of LFPs plotted against the number of PDSs for each simultaneous whole-cell and field recording with the calculated Deming regression line plotted. The correlation between the number of LFPs and the PDSs is significant (p = 0.0028, n = 7, r = 0.9643) and the calculated regression line intersects the x-axis at 10.5.

To verify that PDSs recorded in whole-cell mode in dentate granule cells reflected on-going epileptiform activity in the dentate gyrus, field postsynaptic potentials were recorded simultaneously with the whole-cell patch-clamp recordings in eight of the 21 slices, (three with NPY application and five with NPY + BIIE0246 application). Results showed that field epileptiform discharges in the granule cell layer corresponded with the PDS-events observed in whole-cell patch-clamp recordings (Fig. 5). There was a high degree of correlation between the number of PDSs in individual neurons and respective field potential discharges (r = 0.9643, p = 0.0028. n = 7, Spearman correction, Fig. 5a,d). Moreover, cross-correlation analysis revealed a time-locked temporal relationship between the PDSs in individual neurons and field discharges (Fig. 5b,c). In all three slices with NPY application, a decrease in the number of field epileptiform bursts was observed (139.7 ± 87.04 bursts during *baseline* and 50 ± 22.28 bursts during *NPY*, *n* = *3*). In slices with NPY + BIIE0246 application, there was no overall average decrease in number of bursts during the application period (17 ± 12.03 bursts during *baseline* and 22 ± 14.54 bursts during *NPY* + *BIIE0246*, *n* = *5*). These data support that PDSs recorded in individual neurons corresponded to epileptiform discharges in the dentate gyrus, thereby providing a reliable read-out for experimental outcomes, and suggesting suppression of epileptiform activity in dentate gyrus by NPY.

Taken together, our results show that application of NPY significantly reduces on-going epileptiform activity in dentate granule cells of human hippocampal slices obtained from drug-resistant patients with TLE. The anti-epileptiform effect of NPY was no longer observed in the presence of the NPY Y2 receptor antagonist BIIE0246, suggesting that the Y2 receptor is mediating this NPY effect, most likely by decreasing glutamate release.

## Discussion

We demonstrate that NPY application, in hippocampal slices surgically resected from patients with drug-resistant TLE, significantly reduces chemically induced epileptiform activity in the dentate gyrus. This novel finding suggests that in epileptic brain tissue, where conventional AEDs are ineffective, increasing the levels of NPY could be an alternative approach to achieve a therapeutic effect and suppress seizure activity.

Hippocampal slices from patients with drug-resistant TLE are emerging as an important *in vitro* platform to test new promising treatments established in animal models. It is highly warranted to test novel treatments for efficacy in human brain tissue resistant to conventional AEDs^22,23^. The positive outcome in epileptic resected tissue would increase the likelihood that the approach will be replicated in patients with drug-resistant epilepsy.

The 4-AP-*in vitro* model is widely used to induce epileptiform activity in the rodent as well as human temporal lobe slices^24–26^. 4-AP mainly acts by blocking voltage-gated potassium channels of the K_v_-1 family, thereby broadening APs and leading to increased glutamate and GABA-release^27–29^. In previous studies, 4-AP application alone was unable to initiate interictal epileptiform activity in the human dentate gyrus^22,30^. However, when combined with low Mg^2+^ and/or high potassium, 4-AP induced stable interictal epileptiform bursting in the dentate gyrus^24^ observed with field recordings.

The effect of NPY on seizure-like activity was evaluated by whole-cell patch-clamp recordings of dentate granule cells, where effect on epileptiform activity was assessed by quantification of PDSs, cumulative probability of inter-spike intervals, plotted over time, as well as overall number of APs (the latter as a relative measure of cellular excitability). In all of the above-mentioned parameters we observed statistically significant changes during NPY application, indicating decreased excitability. Since we also demonstrate, as in several previous studies^31–33^, the correlation between PDSs in individual neurons with field discharges from the same area, one could conclude that NPY application has an inhibitory effect on epileptiform activity in the dentate gyrus of drug-resistant human epileptic tissue.

These *in vitro* findings in human hippocampal slices could be extrapolated, with certain degree of confidence, to the human brain *in vivo*. Indeed, there is a strong correlation between what has been observed in *in vitro* animal models of epileptiform activity with *in vivo* (both acute and chronic) epilepsy models in terms of NPY-mediated inhibition of seizure activity. We and others have shown that application of NPY onto rodent hippocampal slices has profound inhibitory effect on epileptiform activity induced by various means, including 4-AP^10–14^. These findings were validated and confirmed *in vivo*: It has been shown e.g. that overexpression of NPY bilaterally in the hippocampus significantly attenuates acute status epilepticus^9^, as well as decreases the SRSs after status epilepticus^34^. We have also shown that combined overexpression of NPY and its Y2 receptor bilaterally in the hippocampus has seizure-suppressant effect in electrical kindling and systemic Kainate models of acute seizures^21^. This seizure-suppressant effect in animals has been shown to be mediated mostly via activation of Y2 receptors^15,21^. Moreover, we have recently demonstrated that simultaneous unilateral overexpression of NPY and its Y2 receptor in the hippocampus has a disease-modifying effect, halting and even reversing the progressive increase of SRS frequency in the post-status epilepticus chronic epilepsy model (intrahippocampal kainate model)^11^. Thus, akin to rodent data our present *in vitro* study in human hippocampal slices predict that NPY might also exert its seizure-inhibitory effect in human chronic TLE. This hypothesis was supported by our previous study showing that NPY acting on Y2 receptors is responsible for attenuation of excitatory synaptic transmission in human drug-resistant epileptic hippocampal slices^6^. The important role of the Y2 receptors in suppressing seizure-like activity in human hippocampal tissue is now demonstrated for the first time in the present study.

What is the possible mechanism of seizure-suppressant effect of NPY in human epileptic hippocampal slices? One explanation would be analogous to what has been proposed in rodent studies: exogenous NPY acts via activation of Y2 receptors located on the presynaptic terminals of excitatory glutamatergic synapses to decrease glutamate release by negatively regulating voltage-gated calcium channels^15,17^. This ultimately leads to attenuation of epileptiform activity, which often is driven by excitatory glutamatergic transmission, particularly the fast interictal-like bursts in the 4-AP model^27^. Similarly, interictal discharges in human epileptic (subicular) slices has been shown to depend on glutamatergic (as well as GABAergic) signalling^35^. Our previous findings in human epileptic hippocampus demonstrate significant reduction of excitatory postsynaptic potentials in principal neurons by NPY application^6^. This effect was dependent on activation of presynaptic Y2 receptors on glutamatergic synapses, since addition of the specific Y2 receptor antagonist BIIE0246 blocked the effect^6^. In the same study, we also demonstrated that Y2 receptors are present and functional in human epileptic hippocampal tissue further supporting that in human epileptic brain tissue, NPY suppress epileptiform activity by decreasing glutamate release from excitatory presynaptic terminals.

The magnitude of the NPY effect in human slices was quite pronounced, showing on average 60% reduction in all but one of the parameters utilised to characterise epileptiform activity. Epileptiform activity, however, was not stopped completely. A possible explanation for this incomplete inhibition could be that 4-AP increases neurotransmitter release from both glutamatergic and GABAergic presynaptic terminals^27^, were GABA may provide depolarising synaptic inputs to principal neurons when altered distribution of Cl^−^ ions across the membrane lead to reversal of chloride currents^36,37^ (reviewed by^38^). If one assumes that NPY predominantly affects glutamatergic inputs, the GABAergic inputs may still be responsible for driving remaining epileptiform activity.

Another interpretation could be that the partial effect of NPY is due its insufficient concentration within slices and/or in the perfusion medium. Indeed, we have observed only partial blockade of excitatory synaptic transmission both in dentate gyrus and in CA1 of the human hippocampus by the same NPY concentration in the aCSF^6^. One could speculate that an increasing NPY concentration in the perfusion medium may have more pronounced inhibitory effect on epileptiform activity.

In translationally designed animal experiments, reflecting possible future clinical scenarios, NPY/Y2 receptor based gene therapy has been shown to inhibit SRSs and therefore proposed as an alternative treatment strategy for focal epilepsies, particularly drug-resistant epilepsies, such as in patients undergoing resective surgery^11^. In this context, the present study takes a major decision-making step for a future gene therapy approach when demonstrating for the first time that NPY is able to suppress epileptiform activity in drug-resistant epileptic tissue from patients with TLE. Lastly, this study underscores the value of the unique possibility provided by epilepsy surgery to validate novel treatment strategies in human drug-resistant epileptic tissue, thereby potentially minimising the risks for failure when proceeding to costly and time-consuming clinical trials (Fig. 1).

## Methods

### Study design

The aim of this study was to test if NPY can have a seizure-suppressant effect in human hippocampal tissue, resected from patients with drug-resistant epilepsy. For the whole study, we collected hippocampal tissue from 10 patients (eight from Copenhagen and two from Lund) undergoing surgical treatment for drug-resistant epilepsy between years 2016 and 2017 (Table 1). The use of tissue from epilepsy surgery patients for research were approved by the local Ethical Committee in Copenhagen (H-2-2011-104) and Lund (#212/2007) and was performed in accordance with the Declaration of Helsinki. Written informed consent was obtained from all subjects prior to each surgery. First, epileptiform activity was induced with [0Mg^2+^]/4-AP-aCSF and recorded with whole-cell patch-clamp technique on granule cells in the dentate gyrus. Subsequently, NPY was added to the [0Mg^2+^]/4-AP-aCSF, and changes in epileptiform activity were recorded and later analysed. To further investigate the mechanism behind the anti-seizure effect of NPY we focused on the Y2 receptor. In these experiments, epileptiform activity was induced as before but the Y2 receptor antagonist BIIE0246 was added together with NPY. Post-surgical morphological evaluation of the hippocampus was performed by a pathologist at the respective hospitals, and diagnosis for hippocampal sclerosis was determined according to ILAE guidelines^39^.

### Slicing and incubation

A detailed description of the methods for slicing and incubation of human brain tissue in the interface chamber can be found in our previous publication^40^. In short, we transported the tissue from the operating room to the electrophysiology laboratory in an ice-cold sucrose-based slush containing in mM: 200 sucrose, 21 NaHCO_3_, 10 glucose, 3 KCl, 1.25 NaH_2_PO_4_, 1.6 CaCl_2_, 2 MgCl_2_, 2 MgSO_4_ (all from Sigma-Aldrich, Sweden), adjusted to 300–310 mOsm, pH 7.4. At the laboratory, the tissue was then transferred into the same type of sucrose slush, continuously bubbled with 95% O_2_ and 5% CO_2_. The tissue, as a whole or parts of the tissue, was then glued onto the cutting platform and placed in a vibratome (VT1200, Leica Microsystems). The hippocampus was sliced in the coronal direction with a slice thickness of 400 µm, while submerged in the sucrose-based slush. When cut, the slices rested fully submerged in a pre-incubation bath with 34 °C aCSF containing in mM: 129 NaCl, 21 NaHCO_3_, 10 glucose, 3 KCl, 1.25 NaH_2_PO_4_, 2 MgSO_4_, and 1.6 CaCl_2_, adjusted to 300–310 mOsm, pH 7.4 for 15–30 min before they were transferred to the interface incubation chamber. The closed interface incubation chamber contained humidified air, and the slices rested on cell culture insets (Millicell) fixed in a smaller open box with a constant flow of carbonated aCSF. The slices were incubated for approximately 24 hours in the interface chamber before electrophysiology recordings.

### Electrophysiology

Slices were individually transferred to a dual-flow recording chamber and held in place by a horseshoe shaped flattened platinum wire. The slices were placed on a metal grid perfused with preheated 32 °C oxygenated aCSF with laminar flow directed above and below the slice at a flow rate of 2 ml/min/channel. Whole-cell recordings were performed from granule cells in the dentate gyrus with glass capillaries containing a AgCl-electrode, with a tip resistance between 2.5 and 6 MΩ when backfilled with a solution containing in mM: 122.5 K-gluconate, 12.5 KCl, 10 KOH-HEPES, 0.2 KOH-EGTA, 2 Mg-ATP, 0.3 Na_3_GTP, and 8 NaCl, pH 7.2–7.4 (mOsm 290–300). Pipettes were connected to a HEKA amplifier (HEKA, Germany) controlled with HEKA Patchmaster software. After formation of a Giga-seal, the patch was ruptured and whole-cell current-clamp recordings were performed. In some cases, field recordings in the granule cell layer, at least 500 µm apart from the patched granule cell, were simultaneously performed using glass capillaries with a tip resistance between 1 and 2 MΩ when backfilled with aCSF.

### Epileptiform activity

In 31 hippocampal slices, 20 out of 39 whole-cell recorded dentate granule cells displayed stable epileptiform activity induced by [0Mg^2+^]/4-AP-aCSF. In these cases, application of NPY or NPY + BIIE0246 (a selective Y2 receptor blocker) was performed. In 12 of these cells, the recording was stable enough to also allow for their monitoring during the washout period. In eight of the 20 slices, field potentials and whole-cell recordings were simultaneously performed, with field electrodes positioned in the granule cell layer. Cells were recorded in current-clamp mode to monitor baseline activity for at least 15 min in normal aCSF, before switching to [0Mg^2+^]/4-AP-aCSF. The [0Mg^2+^]/4-AP-aCSF was prepared in the same way as normal aCSF but omitting Mg^2+^ and adding 4-AP, a blocker of the Kv1-family voltage-gated potassium channels, at a final concentration of 100 µM. A baseline recording with robust epileptiform activity in current clamp-mode was recorded for at least 10 min before applying NPY.

### Administration of NPY and Y2 receptor antagonist

After baseline recordings in [0Mg^2+^]/4-AP-aCSF, the perfusion solution was switched to NPY-containing [0Mg^2+^]/4-AP-aCSF, in which C-terminally amidated NPY (dissolved in dH_2_O, batch: 16-05-2016, Schafer-N, Denmark) was added to a final concentration of 1 µM, all in a siliconized bottle to prevent NPY adhesion to the container walls. Continuous current-clamp recording was performed for 10 min, while perfusing slices with NPY-[0Mg^2+^]/4-AP-aCSF, followed by a washout period, in [0Mg^2+^]/4-AP-aCSF. In a subset of recordings, the Y2 receptor antagonist (BIIE0246, first dissolved in ethanol (0.25 mM) then dH_2_O, Tocris Bioscience, UK) was added to the NPY-[0Mg^2+^]/4-AP-aCSF to a final concentration of 0.6 µM.

### Immunohistochemistry

Biocytin (Sigma-Aldrich) was added to the intracellular solution to label recorded cells and after completion of electrophysiological recordings, the slices were fixed in 4% paraformaldehyde (PFA) in phosphate buffered saline (PBS) overnight and then stored submerged in Walter’s antifreeze solution (ethylene glycol and glycerol in PBS) at −20 °C. After rinsing the slices in potassium-PBS (KPBS), a 1-hour incubation in blocking solution containing 5% normal goat serum (NGS) and triton-KPBS (TKPBS) was followed by 48 hours incubation (at −4 °C) with mouse-MAP2 (1:200 Sigma, M2320) as primary antibody in 5% NGS. Slices were rinsed (3 × 10 min) with TKPBS followed by incubation with mouse-Cy3 (1:400 Jackson ImmunoResearch, 115-165-003) and streptavidin-Alexa488 (1:400, Invitrogen, S11223) as secondary antibodies in 5% NGS for 2 hours, rinsed (3 × 10 min) with KPBS and then mounted on glass slides and cover slipped with DABCO.

### Data analysis and statistics

Electrophysiological recordings were analysed in IGOR Pro version 6.3 (Wavemetrics) and Minianalysis (Synaptosoft), while Prism (GraphPad) was used for further statistical analysis. The last 300 seconds of *baseline*, *NPY, NPY* + *BIIE0246* and *washout* recordings were compared in the evaluation of epileptiform activity. In the first part of the analysis, Minianalysis and Prism were used to calculate the intervals between APs and to generate a cumulative plot on which the Kolmogorov-Smirnov test was applied to test if the plotted curves were significantly separated from each other. Further analysis of the recordings included counting of APs and PDSs. The APs were automatically detected by the Minianalysis software, using a detection threshold of 15 mV above baseline. The PDS were detected manually, defined by a depolarising ridge, which is not disrupted by repolarisation between APs, with the largest PDS in the baseline recording as a standard giving the inclusion parameter of 50% of largest PDS amplitude. All PDS with lower amplitude than 50% of the largest PDS in the baseline recording were excluded from analysis. The Ratio-based Paired t-test was used to compare the data from the baseline recording with the wash-in period of either NPY or NPY + BIIE0246. Differences between groups were considered statistically significant when p < 0.05. The correlation analysis between number field potentials and PDSs from the simultaneous whole-cell and field recordings were done with Spearman correction. The Deming regression line was calculated using the standard deviation from each group (number of field potentials, number of PDS). Cross-correlation analysis was performed in Clampfit software (Molecular Devices) using a 1-second sliding window across 10 minutes of recording. Traces from both the whole-cell and field recordings were filtered with two iterations of low-pass filtering at 40 Hz before the analysis, to reduce the contribution of action potentials to the shape of individual PDS.

## Data Availability

The datasets generated and/or analysed during the current study are available from the corresponding author on reasonable request.
